# Molecular investigation of proteinase inhibitor (PI) gene in tomato plants induced by Meloidogyne species

**DOI:** 10.1186/s43141-021-00230-2

**Published:** 2021-08-30

**Authors:** Refik Bozbuga

**Affiliations:** Biological Control Research Institute, Nematology Lab, 01321, Yuregir, Adana, Turkey

**Keywords:** Gene expression, Proteinase inhibitors I gene, nematode, Tomato, *Meloidogyne incognita*, *Meloidogyne javanica*, *Meloidogyne chitwoodi*

## Abstract

**Background:**

The plant parasitic nematode genus *Meloidogyne* parasitize almost all flowering crops. Plants respond with a variety of morphological and molecular mechanisms to reduce the effects of pathogens. Proteinase inhibitors (PI), a special group of plant proteins which are small proteins, involve in protective role in the plants attacked by microorganisms. Still, the plant response using PI against nematodes has not been well understood. Therefore, this study was aimed to determine the expression of proteinase inhibitor I (PI-I) gene subsequent the infection of *M. incognita*, *M. javanica*, and *M. chitwoodi* in tomato plants post nematode infections. Molecular methods were used to determine the *PI* gene expressions at different days post nematode infections in host tissues.

**Results:**

Results revealed that the population of *M. incognita* species reached the highest level of nematode population followed by *M. javanica* and *M. chitwoodi,* respectively. All Meloidogyne species induced expression of PI-I gene reached at the utmost level at 3 days post infection (dpi) in host tissues. Relative gene expression level was sharply dropped at 7 dpi, 14 dpi, and 21 dpi in *M. incognita* induced gene expression in host tissues. Similar results were observed in host tissues after infection of *M. javanica* and *M. chitwoodi.*

**Conclusions:**

The commonalities of plant response across a diverse Meloidogyne species interaction and the expression of *PI gene* may be related to plant defense system. Increased level of *PI* gene expressions in early infection days in host tissues induced by parasitic nematodes may share resemblances to the mechanisms of resistance on biotrophic interactions.

## Background

Plant cell wall is important component in plant cell and plays an essential role against pathogens [1, 2]. The modification of plant cell wall molecular architecture occurs during the nematode infection that presence and distribution of glycoproteins, pectin, and hemicellulose-related polysaccharides have been changed in nematode feeding site [3]. A parasitic nematode group, Meloidogyne genus termed also root knot nematodes, creates a unique feeding site termed giant cell to supply nutrient to the nematode from plant [4]. The cell wall also has physical barriers with cellulose, hemicellulose, lignin, proteins, and chemical substances [5]. The thickness of giant cell walls are around 6 times thicker than neighboring cells walls in the nematode feeding site [4]. In addition, gall thickness also varies in different host roots. *M. incognita* caused gall thickness as a percentage of adjacent root thickness are 300%, 250%, 650%, and 250% thicker in maize, Arabidopsis, Aduki bean, and potato, respectively [4].

Plants give response to the nematode infection in molecular level [6–8] that proteinase inhibitors are a group of proteins that demonstrating a generally part inside the plant defense against plant feeders from insects to microorganisms. The proteinase inhibitors involve in perturbing the enzymatic capability in microbic chemicals that obtainable at intervals plant pests; hence, they might not digest plant tissues [9]. Additionally, some proteinase inhibitors involve in plant antimicrobial properties giving limitation of pathogen growth [10]. Protein inhibitors are induced mostly during wounding or chemical signalling through molecules of the plant [11]. Signal molecules involve in production and relocation of proteinase inhibitors by the phloem and xylem of the plant [11]. When an insect feed on plant, those inhibitors involve in less digestion in insect, thus the pest may not grow proper level [12].

Meloidogyne species are most important plant parasitic nematodes species causing economic losses on crops more than a 170 billion dollars in the world [13]. Around a hundred species of root-knot nematodes have been described in the world [14]. Of these species, *M. incognita*, *M. javanica*, and *M. arenaria* are tropical, *M. hapla*, *M. chitwoodi*, and *M. fallax* are temperate species [15]. *M. incognita, M. javanica*, *M. hapla*, and *M. arenaria* are frequently found in many countries and they are called major species [16]. Meloidogyne species are obligate sedentary endoparasites and infect plant roots. The second stage of Meloidogyne species are infective stage and enter plant roots just behind root elongation zone and move intercellularly and create a feeding site [4, 16]. Nematode secretes effectors which are produced in gland cells and cause the formation of feeding sites [17]. Following the starting the feeding of J2s, galls are seen within days [18, 19]. Nematode undergoes third and four stages and finally becomes an adult female. The male may leave from roots (mobile), and the adult female become sedentary and starts to produce eggs [16]. *M. incognita* hatching number of *M. incognita* increases between 25 and 35° but decreases in higher temperatures above 35 °C [20].

The generation of proteinase inhibitors reveals that plants have the capacity to modify their defense behavior in reaction to plant invaders including nematodes. This complex defense mechanism serves to protected plant from pathogens. The expression of PI*-*I gene during the three nematode species has been fully understood. Therefore, this study was aimed to determine *PI* gene expression at 1, 3, 7, 14, and 21 days post-infection of three different root knot nematodes (*M. incognita*, *M. javanica*, *M. chitwoodi*) in tomato plants.

## Methods

### Plant and nematode culture

Susceptible tomato (*Solanum lycopersicon*) plants were used in this experiment. The 25-day-old tomato seedlings were sowed in the 1 kg pots of sterilized soil medium. Soil medium was consisted of 80% sand + 20% garden soil. Soil medium was autoclaved to kill weed seeds, pathogen, and other microorganism in pressure sterilizer at 126 °C for an hour. This study was performed in the greenhouse. The greenhouse temperature was set up as 25 ± 2 °C in 16 h light 8 h dark conditions with 60% of humidity conditions. When tomato seedling roots reached to the bottom of the pot, seedlings were ready to inoculate the nematodes, *Meloidogyne incognita*, *Meloidogyne javanica*, and *Meloidogy*ne *chitwoodi*. The experiment was set up with five repetitions as infected and uninfected plants. Randomized complete block design for each of the experiment was set up with five replications that each plant was grown in separate pots. Two independent experiments were achieved.

The pure nematode cultures of three Root knot nematodes species, *Meloidogyne incognita*, *Meloidogyne javanica*, and *Meloidogy*ne *chitwoodi*, which already exist in the greenhouse, were used for this experiment. To maintain the nematode population, pure culture of second stage juveniles were given to susceptible tomato plants at 25 ± 2 °C at %60 humidity and 16 h light and 8 h dark conditions. Hatching was performed using Modified Baerman funnel technique. The second stage of juveniles (j2) was collected, counted, and optimized. The viability juveniles were checked under the stereo microscope. Then, the nematode infection assay was achieved. The number of second stage of nematode juveniles was calculated as 1 J2s per cm^3^ soil mix (1000 J2s for each pot) for assay. Two centimeters depth of four holes were opened on the on the surface of pots and in total 1000 j2s were given on holes. Pots with plants were placed for 2 months in greenhouses at 25 ± 2 °C. Nematode population was calculated among differences of initial and final nematode population. Following the nematode infection, 2 months later, infected roots with 200 g of soil and root samples with five repetitions for each experiment were taken. Soil and root samples were brought to the nematology lab. Nematode extraction was achieved using modified Baermann funnel method. Second stage juveniles were collected and counted under the stereo microscope. Final number of nematode populations was calculated. Variance analysis (ANOVA) was performed at *P* ≤ 0.05 according to Duncan’s multiple range test to determine the difference among nematode populations.

### Gene expression

Tomato leaves were collected at 1, 3, 7, 14, and 21 dpi following the nematode infections. Three bunch of leaves of five tomato plants from each replicate were placed in liquid nitrogen. GeneJET plant RNA purification mini kit (Thermo Scientific, Lithuania) was used to isolate RNA from plant tissues. Plant RNA lysis Solution was pipetted (500 μL) and into 1.5 mL micro centrifuge tube. Plant tissue was weighed at 100 mg from frozen tissue and grinding the plant tissues using mortar and pestle into the liquid nitrogen. Incubated for 3 min at 56 ^o^C and centrifuged at 14,000 rpm for 5 min. Supernatant was collected and transferred to the clean micro centrifuge tube, added 250 μL 96% ethanol and mixed by using pipet. Prepared mix was transferred to the purification column inserted in a collection tube and centrifuged at 11000 rpm for 1 min. The flow-through solution was discarded, reassembled column and collection tube. Seven hundred micro liters of wash buffer 1 were added to the purification column and centrifuged at 11,000 rpm for 1 min. Flow-through was discarded and purification column were placed into a clean 2 mL collection tube. Later, 500 μL wash buffer 2 was added to purification column and centrifuged at 11,000 rpm for 1 min, and later flow-through solution was discarded. Then, the column re-spanned at 14,000 rpm for 1 min and collection tube was discarded. Purification column was transferred to a RNase-free 1.5 mL collection tube. Fifty microliters of nuclease-free water was added to purification column to elute RNA and centrifuged for 1 min at 11,000 rpm. Purification column was discarded, and purified RNA was used. The amount of RNA was measured in nanodrop (Maestrogen nano, Taiwan) and diluted as 1000 nm. Then, cDNA protocols were achieved. iScript cDNA synthesis kit (Bio-Rad, USA) was used for cDNA study. The 5× i Script Reaction mix (4 μl), iScript Reverse Transcriptase (1 μl), Nuclease free water (14.5 μl), RNA template (0.5 μl) with total 20 μl volume were prepared. The reaction mix was incubated in a thermal cycler using the following reaction: priming for 5 min at 25 ^°^C, reverse transcription 20 min at 46 ^°^C, RT inactivation for 1 min at 95 ^°^C. cDNAs were diluted in 1/10 to use q-PCR studies. Then PCR was performed using SsaAdvanced Universal SYBR green Super mix (Bio-Rad, USA). The component of reaction (Sso advanced universal SYBR green super mix, forward and reverse primers, DNA template, nuclease-free water) was prepared in qPCR tubes.

Primers of PI-I gene sequences: Forward: 5′-TTGCTCTCCTCCTTTTATTTGG-3′; Reverse: 5′-GCAAGCCTTGGCATGTTC-3′ were performed [21, 22]. The *Solanum lycopersicon* actin gene primers (Forward: 5′-ATGTATGTTGCCATCCAGGCT-3′, Reverse: 5′-TGTGGCTGACACGATCTCCA-3′) were used as a housekeeping gene [23]. Actin gene was used as a reference gene for normalizing mRNA levels of target genes following the infection of nematodes: *M. incognita*, *M. javanica*, and *M. chitwoodi*. Thermal cycling protocol on a real-time PCR was achieved. After polymerase activation and DNA denaturation at 95 ^°^C for 30 s, amplifications were performed for 40 cycles at 95 °C for 15 s and at 60 °C for 1 min. To check the specificity of the PCR product, the melting curves were analyzed for each data point. Repetition was performed for three samples for each treatment. Ct values were used to determine the expression levels. Relative gene expression was calculated using the 2^△△^Ct method and uninfected (control) values were subtracted from infected (nematode infected) values.

## Results

Results of this study were given under the two separate subheadings that firstly determine the differences of nematode populations among three nematode populations. Secondly, gene expression of PI-I gene on tomato plants following the infection of *M. incognita*, *M. chitwoodi*, and *M. javanica* at 1, 3, 7, 14, and 21 dpi.

**Fig. 1:**
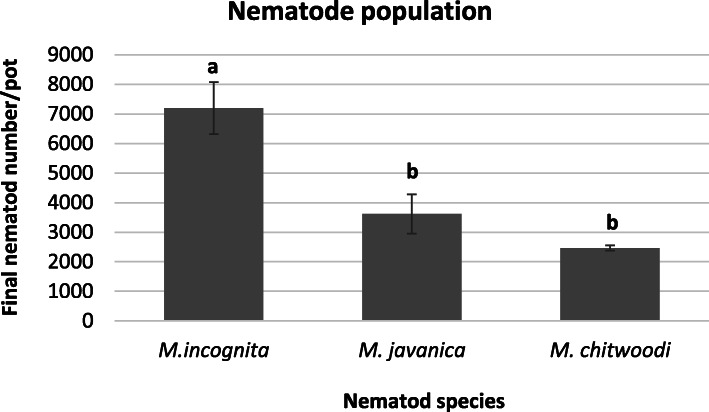
Nematode population (reproduction) of *Meloidogyne* species: *M. incognita*, *M. javanica*, *M. chitwoodi* in the roots of tomato plants. Initial nematode number (first infection number) was 1000 nematode in each pot for each plant. Final nematode number was calculated following the 2 months later of nematode inoculation. Randomised block design was accomplished with five repetitions. *Y* axis represents final nematode number per plant, *X* axis indicates nematode species. Error bars represents the standard error of the means of five replicates. Letters on the column indicates the statistical differences (*P* < 0.05) among nematode populations

Reproduction differences of nematode population on tomato plants: This study was aimed to determine the differences in the reproduction rate of three nematode species in same host plant (tomato). Initial nematode number per pot was 1000 J2s and final population of nematodes were counted 2 months later. Results revealed that the population of all nematode species increased. Highest nematode number in per plant was determined in *M. incognita* species (7200 J2s) that it was statistically grouped differently compared to other species. The nematode number was 3617 J2s and 2467 J2s in *M. javanica* and *M. chitwoodi,* respectively. However, *M. javanica* and *M. chitwoodi* were placed statistically in the same group (Fig. 1). It can be said from the results that *M. incognita* parasites the tomato plant more than others.

**Fig. 2:**
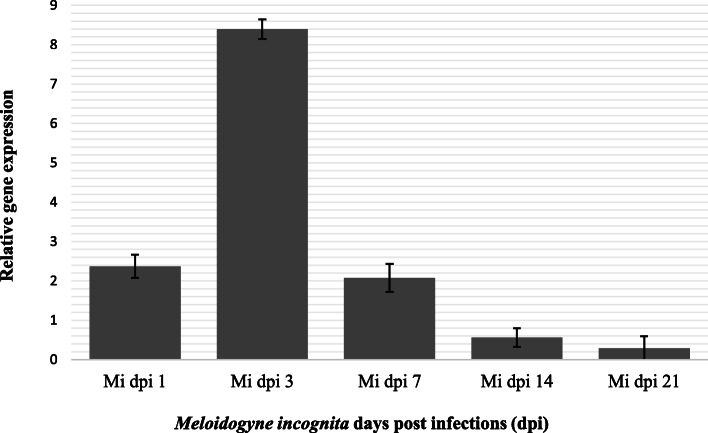
*Meloidogyne incognita* induced proteinase inhibitor I gene expression in host tissues at different days post-infections. Housekeeping gene Actin gene was used to normalize expression of genes of interest. Relative gene expression was calculated biological replicates using the 2^△△Ct^ method and uninfected (control) values were subtracted from infected values. *Y* axis represents relative gene expressions, *X* axis indicates 1 dpi, 3 dpi, 7 dpi, 14 dpi, and 21 dpi following *Meloidogyne incognita* infection. Error bars represents the standard error of the means of three replicates. Mi, *Meloidogyne incognita*; dpi, days post infection

**Fig. 3:**
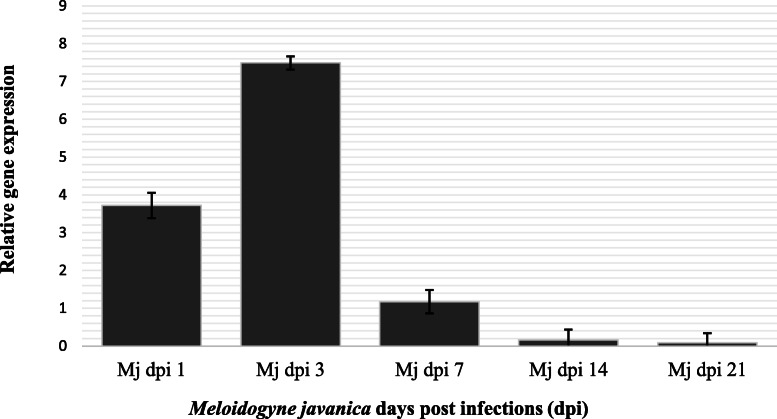
*Meloidogyne javanica* induced *proteinase inhibitor I* gene expression in host tissues at different days post infections. Housekeeping gene actin gene was used to normalize expression of genes of interest. Relative gene expression was calculated biological replicates using the 2^△△Ct^ method and uninfected (control) values were subtracted from infected values. *Y* axis represents relative gene expressions, *X* axis indicates 1 dpi, 3 dpi, 7 dpi, 14 dpi, 21 dpi following *Meloidogyne javanica* infection. Error bars represents the standard error of the means of three replicates. Mj, *Meloidogyne javanica*; dpi, days post infection

**Fig. 4:**
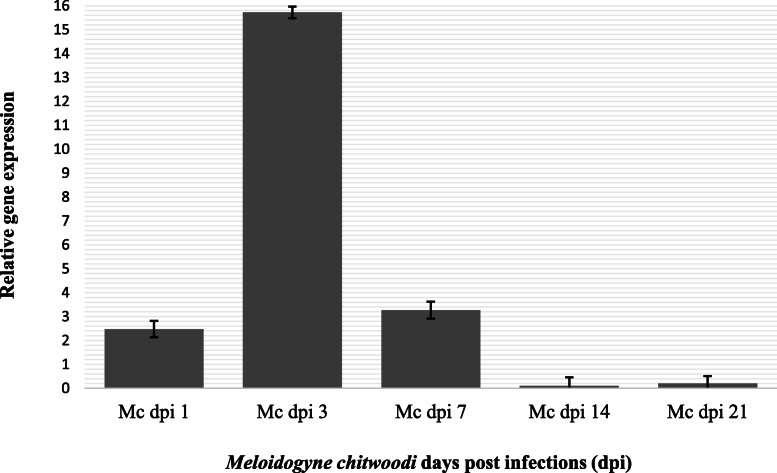
*Meloidogyne chitwoodi* induced *proteinase inhibitor I* gene expression in host tissues at different days post-infections. Housekeeping gene Actin gene was used to normalize expression of genes of interest. Relative gene expression was calculated biological replicates using the 2^△△Ct^ method and uninfected (control) values were subtracted from infected values. *Y* axis represents relative gene expressions, *X* axis indicates 1 dpi, 3 dpi, 7 dpi, 14 dpi, and 21 dpi following *Meloidogyne chitwoodi* infection. Error bars represents the standard error of the means of three replicates. Mc, *Meloidogyne chitwoodi*; dpi, days post infection

Secondly, *proteinase inhibitor I (PI-I) gene* expression on tomato plants following the infection three root knot nematodes were determined. *Meloidogyne incognita* induced *proteinase inhibitor* (*PI*) gene expression levels in host tissues at 1, 3, 7, 14, and 21 dpi were given in Fig. 2. Value of relative gene expression level was 2.37 at 1 dpi then raised and reached the highest peak at 3 dpi (8.39). Value of relative gene expression level sharply dropped as 2.07, 0.56, and 0.29 at 7 dpi, 14 dpi, and 21 dpi, respectively (Fig. 2). *Meloidogyne javanica*-induced proteinase inhibitor (PI) gene expression in host tissues were given in Fig. 3. The value of gene expression was 3.71 in *M. javanica* infected host tissues at 1 dpi. Gene expression level was doubled at 3 dpi following the 1 dpi (Fig. 3). The highest peak was observed at 3 dpi and severely decrease of gene expression value (1.17) was observed at 7 dpi. The lowest values of gene expression following the infection of *M. javanica* were determined 14 and 21 dpis (Fig. 3). Similar trend line was observed in *M. incognita* and *M. javanica* induced PI-I gene expression value in host tissues (Figs. 2 and 3). *Meloidogyne chitwoodi* induced *proteinase inhibitor* (*PI*) gene expression values in host tissues at different post infections were presented in Fig. 4. The values of gene expression were 2.47 at 1 dpi following the infection of *M. chitwoodi*. The gene expression value was observed as 15.72 at 3 dpi. Decreasing value of gene expression was observed at 7 dpi (value 3.21), 14 dpi (value 0.10), and 21 dpi (value 0.20). The peak of the polynomial trendline of the gene expression value was observed at 3 dpi following the infection of *M. chitwoodi* (Fig. 4). The value of gene expression at 3 dpi as the highest peak among not only the *M. chitwoodi* induced gene expression at 1, 7, 14, and 21 dpis but also other species (*M. incognita* and *M. chitwoodi*) induced gene expression values (Figs. 2, 3, and 4). The high level of gene expression at 3 dpis can be related to plant resistance against nematode because of nematode population was lowest in *M. chitwoodi* infection, however gene expression was doubled compared to values of other nematode species (*M. incognita, M. javanica*) induced host plant gene expressions (Figs. 1, 2, 3, and 4). In general, PI-I gene expression was observed as the highest value at 3 dpis at the infection of all nematode species in tomato plants (Figs. 2, 3, and 4). It may conclude that highest gene expression occurs at early dpis.

## Discussion

Protease inhibitors (PIs) are small ubiquitous proteins that they have many biological functions in plants including defense against pathogens [24]. The family of Künitz protease inhibitor amasses when the stress of water occurs in leaves of *Brassica napus* [25]. Kunitz-type potato proteinase inhibitor is localized in the cell wall, plasma membrane of cells, and non-wounded upper leaves in wounded potatoes [26]. PIs are important anti nutritive compounds to defend crop plants from the infection of plant pest or pathogens [27, 28]. Recombinant protease inhibitors are important tools for the development of insect-resistant transgenic crops [29]. Overexpression of cysteine proteinase inhibitor activity involves tolerance to abiotic stress and messenger RNAs are transported to distant tissues of Arabidopsis [30]. Proteinase inhibitors (PIs) are important elements of natural plant defense [31]. In this study, *M. incognita*, *M. chitwoodi*, and *M. javanica* induced gene expressions in tomato plants upregulated at 1 and 3 dpis and decreased at 7, 14, and 21 dpis. The result of this study revealed that PIs possibly involve in defense mechanism; however, later days nematode suppressed the PIs in the plant during the plant nematode compatible interactions. The plant proteases involve in removal of protein damage and plant defense responses. A plant parasitic nematode *Heterodera schactii* caused to decrease of protease activities in infected roots of Arabidopsis plants. AtCYS1, AtCYS5, and AtCYS6 gene expressions increase upon *H. schactii* infection [32].

Potato cysteine proteinase inhibitor is expressed in Phythophthora resistance potato cultivar White Lady. Silencing of Potato cysteine proteinase inhibitor resulted in increase in lesion size and water soaking [33]. The potato protease inhibitor gene plays roles in the cold-induced sweetening of potato tubers by modulating invertase activity [34]. Kunitz-like inhibitors and proteinase inhibitors 1 are abundant in storage organs of potato plants and are upregulated in other tissues in response to biotic and abiotic stress. However, PI expression profiles are not correlated following the infection of a plant parasitic nematode *Glabodera rostochiensis* with the resistance status of the potato genotype [24]. Plant cysteine proteinase inhibitor and a fungal chitinase which are used to transform tomato (*Solanum lycopersicum*) caused to synergistic effect to defense response genes that caused to inhibitory effect on *Meloidogyne incognita* [35]. In this study, plant could not manage suppress the nematode feeding, therefore, this situation may lead to decrease gene expression of late dpis: 7, 14, and 21 dpis (Figs. 2, 3, and 4).

Some species of Root knot nematodes: *M. incognita, M. javanica*, *M. arenaria*, *M. chitwoodi*, *M. fallax*, and *M. hapla* are the most common parasitic nematode species and may found 95% of the globe in crops [36]. Nematodes cause significant yield losses in vegetables worldwide and these losses are 42–54% in tomato [15]. *M. incognita* is placed in the top list among plant parasitic nematodes [14]. The population of *M. incognita* reproduction rate was reached at the highest level comparing other nematode species in this study (Fig. 1).

Plant response with multiple layers of defenses and triggers resistance to pathogens and Salicylic acid involves in defense response [37] and the mechanisms of systemic induced resistance have been studied by many researchers [38–40]. PIs probably involves in the defense mechanisms in early days post infection, because gene expression was reached the highest level at 1 and 3 dpis (Figs. 2, 3, and 4).

## Conclusions

PI-I and II synthesis occur in leaves of wounded tomato plants [41] and PI-I involves in plant defense mechanism. In this study, PI gene involved in plant systemic induced resistance mechanism at early infection time. This revealed that the gene expression of PI shared similarities with the response of tomato to different nematode species. The gene expression commonalities across diverse nematode species in the same host may play an important role for potential wide range of molecular resistance mechanisms.

## Data Availability

All data generated/analyzed during this study are included in this manuscript.

## Abbreviations

cDNA: Complementary DNA
Ct: Cycle threshold
DNA: Deoxyribonucleic acid
dpi: Days post-infection
j2: Second stage of juvenile
mRNA: Messenger RNA,
PCR: Polymerase chain reaction
PI: Proteinase inhibitor
RNA: Ribonucleic acid
SA: Salicylic acid

## References

[1] Caffall KH, Mohnen D (2009). The structure, function, and biosynthesis of plant cell wall pectic polysaccharides. Carbohydr Res.

[2] Albersheim P, Darvill A, Roberts K, Sederoff R, Staehelin A (2011). Plant cell walls: from chemistry to biology.

[3] Bozbuga R, Lilley JL, Knox JP, Urwin PE (2018) Host-specific signatures of the cell wall changes induced by the plant parasitic nematode, *Meloidogyne incognita*, Sci Rep 8(1);17302. 10.1038/s41598-018-35529-7 10.1038/s41598-018-35529-7 PMC6251906 30470775

[4] Bozbuga R (2017). Characterisation of cell walls at the feeding site of *Meloidogyne incognita*, PhD thesis, University of Leeds

[5] Heredia A, Jimenez A, Guillen R (1995). Composition of plant-cell walls. Zeitschrift Fur Lebensmittel-Untersuchung Und-Forschung.

[6] Bozbuga R (2020). Expressions of *Pathogenesis related 1* (*PR1*) Gene in *Solanum lycopersicum* and Influence of Salicylic Acid Exposures on Host-*Meloidogyne incognita* Interactions. Dokl Biochem Biophys.

[7] Bozbuga R (2020). Effect of Submerging *Solanum lycopersicum* Roots in Salicylic Acid (SA) Solution for Different Durations on Nematode Infection and Expressions of SlPR5 Gene. Horticultural studies.

[8] Bozbuga R (2020). Genetics, molecular interactions and resistance response of common bean (*Phaseolus vulgaris* L.) Genotypes to Root Knot Nematodes (*Meloidogyne* spp). Cutting-Edge Research in Agricultural Sciences.

[9] Shu-Guo F, Guo-Jiang W (2005). Characteristics of plant proteinase inhibitors and their applications in combating phytophagous insects. Botanical Bull Academia Sinica.

[10] Kim JY, Park SC, Hwang I, Cheong H, Nah JW, Hahm KS, Park Y (2009). Protease inhibitors from plants with antimicrobial activity. Int J Mol Sci.

[11] Farmer EE, Ryan CA (1990) Interplant communication: airborne methyl jasmonate induces synthesis of proteinase inhibitors in plant leaves. Proc National Acad Sci 87(19):7713–7716*.* Bibcode*:* 1990PNAS...87.7713F*.*10.1073/pnas.87.19.7713. PMC 54818 10.1073/pnas.87.19.7713 PMC54818 11607107

[12] *Lawrence P*, *Koundal K* (2002) Plant protease inhibitors in control of phytophagous insects. Plant Biotechnology. 5(1). 10.2225/vol5-issue1-fulltext-3*– via* Electronic Journal of Biotechnology.

[13] Elling AA (2013). Major emerging problems with minor Meloidogyne species. Phytopathology.

[14] Jones JT, Haegeman A, Danchin EGJ, Gaur HS, Helder J, Jones MGK, Kikuchi T, Manzanilla-Lopez R, Palomares-Rius JE, Wesemael WML, Perry RN (2013). Top 10 plant-parasitic nematodes in molecular plant pathology. Mol Plant Pathol.

[15] Netscher C, Sikora RA (1990) Nematode parasites on vegetables. Editted, Luc M, Sikora RA, Bridge J Plant Parasitic Nematodes in Subtropical and Tropical Agriculture. CAB International, pp 231-283

[16] Moens M, Perry RN, Starr JL (2010) Meloidogyne species - a diverse group of novel and important plant parasites. Root-Knot Nematodes 1–17

[17] Hewezi T, Howe P, Maier TR, Hussey RS, Mitchum MG, Davis EL, Baum TJ (2008). Cellulose binding protein from the parasitic nematode *Heterodera schachtii* interacts with arabidopsis pectin methylesterase: Cooperative cell wall modification during parasitism. Plant Cell.

[18] Bozbuga R, Dasgan HY, Akhoundnejad Y, Imren M, Toktay H, Kasapoglu EB (2015) Identification of common bean (*Phaseolus vulgaris*) genotypes having resistance against root knot nematode *Meloidogyne incognita*, Legume Res, 38: 669-674. DOI: 10.18805/lr.v38i5.5948

[19] Bozbuga R, Imren M, Kasapoğlu EB, Toktay H, Elekcioğlu IH (2015). Determining the Optimal Meloidogyne Incognita Inoculum Level, Inoculation Time, Pathogencity and Gall Development on Tomato Roots for Resistance Experiments in Breeding Programs. Vegetos.

[20] Toktay H, Bozbuğa R, İmren M, Kasapoğlu EB, Elekcioğlu İH (2014). *Meloidogyne incognita (*Kofoid & White, 1919) Chitwood ve *Meloidogyne hapla* (Chitwood, 1949) (Nemata: Meloidogynidae) Yumurtalarının Açılmasına Farklı Uygulamaların Etkisi ve İkinci Dönem Larvalarının Beslenmeden Yaşayabilme Süreleri (in Turkish). Türk Tarım ve Doğa Bilimleri Dergisi.

[21] Young RJ, Scheuring CF, Harris-Haller L, Taylor BH (1994) An auxin-inducible proteinase inhibitor gene from tomato. Plant Physiol. 104, 2, 811, 812. 10.1104/pp.104.2.811 10.1104/pp.104.2.811 PMC159268 8159801

[22] Campos ML, de Almeida M, Rossi ML, Martinelli AP, Junior CGL, Figueira A, Rampelotti-Ferreira FT, Vendramim JD, Benedito VA, Peres LEP (2009). Brassinosteroids interact negatively with jasmonates in the formation of anti-herbivory traits in tomato. J Exp Bot.

[23] Chinnapandi B, Bucki P, Miyara SB (2017) SlWRKY45, nematode-responsive tomato *WRKY* gene, enhances susceptibility to the root knot nematode; *M. javanica* infection, Plant Signal Behav 2017;12, 12, e1356530. 10.1080/15592324.2017.1356530 10.1080/15592324.2017.1356530 PMC5792125 29271721

[24] Turra D, Bellin D, Lorito M, Gebhardt C (2009). Genotype-dependent expression of specific members of potato protease inhibitor gene families in different tissues and in response to wounding and nematode infection. J Plant Physiol.

[25] Downing WL, Mauxion F, Fauvarque MO, Reviron MP, de Vienne D, Vartanian N, Giraudat J (1992). A Brassica napus transcript encoding a protein related to the Kunitz protease inhibitor family accumulates upon water stress in leaves, not in seeds. Plant J.

[26] Suh SG, Cho JK, Suh EJ, Chung HD, Hannapel DJ (1999). Immunocytochemical intracellular localization of the 22-ku Kunitz-type potato proteinase inhibitor in potato tubers and leaves. J Plant Physiol.

[27] Michaud D (2000) Recombinant protease inhibitors in plants. Georgetown, Landes Bioscience. 241 pages, ISBN 1 58706 007 8

[28] Haq SK, Atif SM, Khan RH (2004). Protein proteinase inhibitor genes in combat against insects, pests, and pathogens: natural and engineered phytoprotection. Arch Biochem Biophys.

[29] Khalf M, Goulet C, Vorster J, Brunelle F, Anguenot R, Fliss I, Michaud D (2010). Tubers from potato lines expressing a tomato Kunitz protease inhibitor are substantially equivalent to parental and transgenic controls. Plant Biotechnol J.

[30] Thieme CJ, Rojas-Triana M, Stecyk E, Schudoma C, Zhang W, Yang L, Minambres M, Walther D, Schulze WX, Paz-Ares J, Scheible WR, Kragler F (2015). Endogenous Arabidopsis messenger RNAs transported to distant tissues. Nature Plants.

[31] Ryan CA (1990). Protease inhibitors in plants: genes for improving defenses against insects and pathogens. Annu Rev Phytopathol.

[32] Labudda M, Różańska E, Szewińska J, Sobczak M, Dzik JM (2016). Protease activity and phytocystatin expression in Arabidopsis thaliana upon *Heterodera schachtii* infection. Plant Physiol Biochem.

[33] El-Banna A, Taller J (2017). Functional characterization of the silenced potato cysteine proteinase inhibitor gene (PCPI) in Phytophthora infestans resistance. Physiol Mol Plant Pathol.

[34] Liu X, Cheng S, Liu J, Ou Y, Song B, Zhang C, Lin Y, Li XQ, Xie C (2013). The potato protease inhibitor gene, St-Inh, plays roles in the cold-induced sweetening of potato tubers by modulating invertase activity. Postharvest Biol Technol.

[35] Chan YL, Yang AH, Chen JT, Yeh KW, Chan MT (2010). Heterologous expression of taro cystatin protects transgenic tomato against *Meloidogyne incognita* infection by means of interfering sex determination and suppressing gall formation. Plant Cell Rep.

[36] Trudgill DL, Blok VC (2001). Apomictic polyphagous root knot nematodes: exceptionally successful and damaging biotrophic root pathogens. Annu Rev Phytopathol.

[37] Nishimura MT, Dangl JL (2010) Arabidopsis and the plant immune system. Plant J 61:1053-1066. 10.1111/j.1365-313X.2010.04131.x 10.1111/j.1365-313X.2010.04131.x PMC2859471 20409278

[38] Sticher L, Mauch-Mani B, Metraux JP (1997). Systemic acquired resistance. Annu Rev Phytopathol.

[39] Pieters CMJ, Van Loon LC (1999). Salicylic acid-independent plant defence pathways. Trends Plant Sci.

[40] Durrant WE, Dong X (2004). Systemic acquired resistance. Annu Rev Phytopathol.

[41] Graham JS, Pearce G, Merryweather J, Titani K, Ericsson LH, Ryan CA (1985) Wound-induced proteinase inhibitors from tomato leaves. II. The cDNA-deduced primary structure of pre-inhibitor II. J Biol Chem 10;260(11):6561-4. PMID: 3838986 3838986

